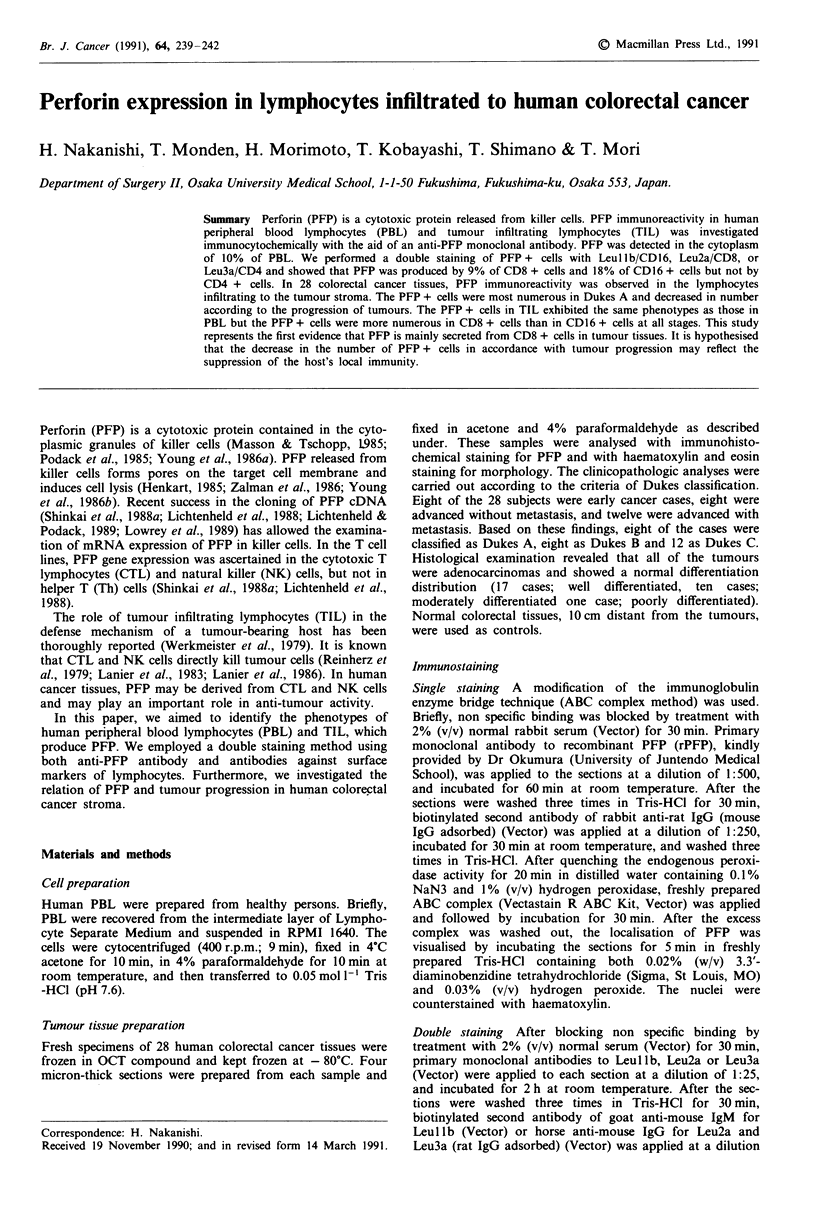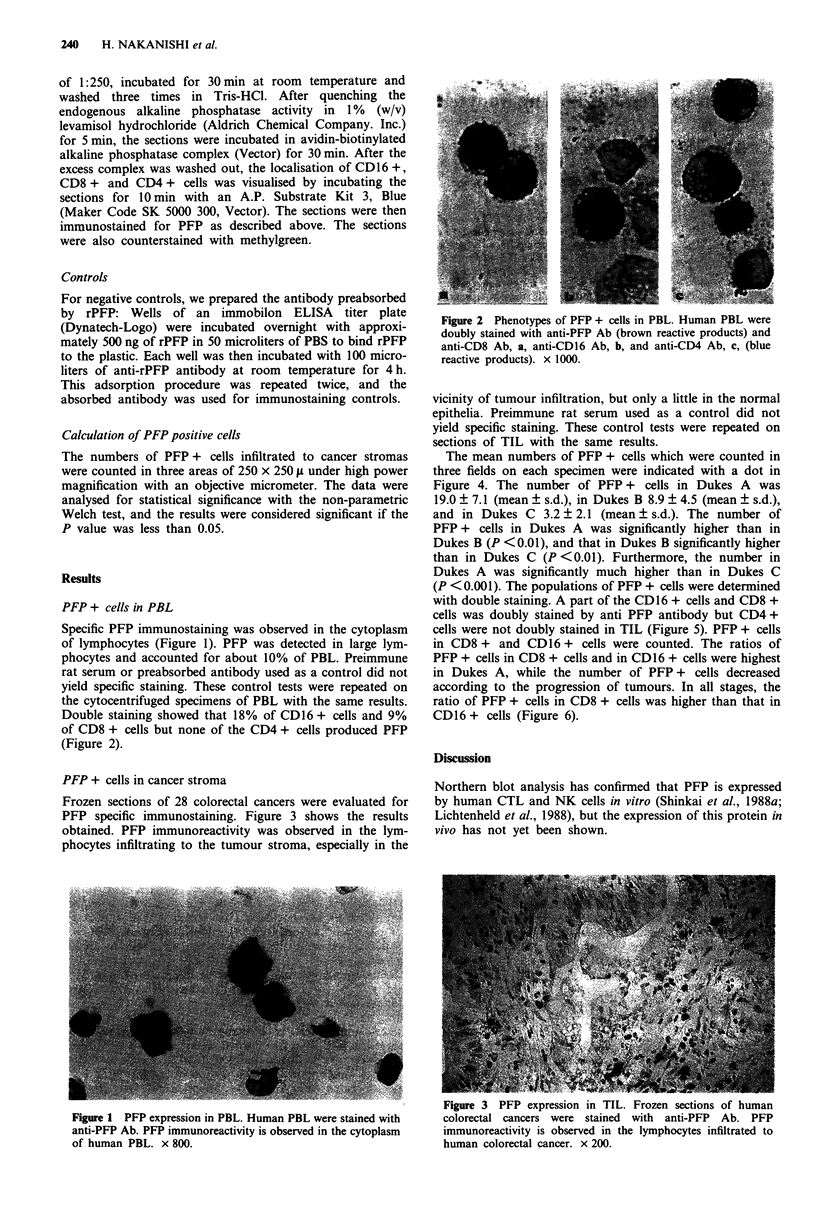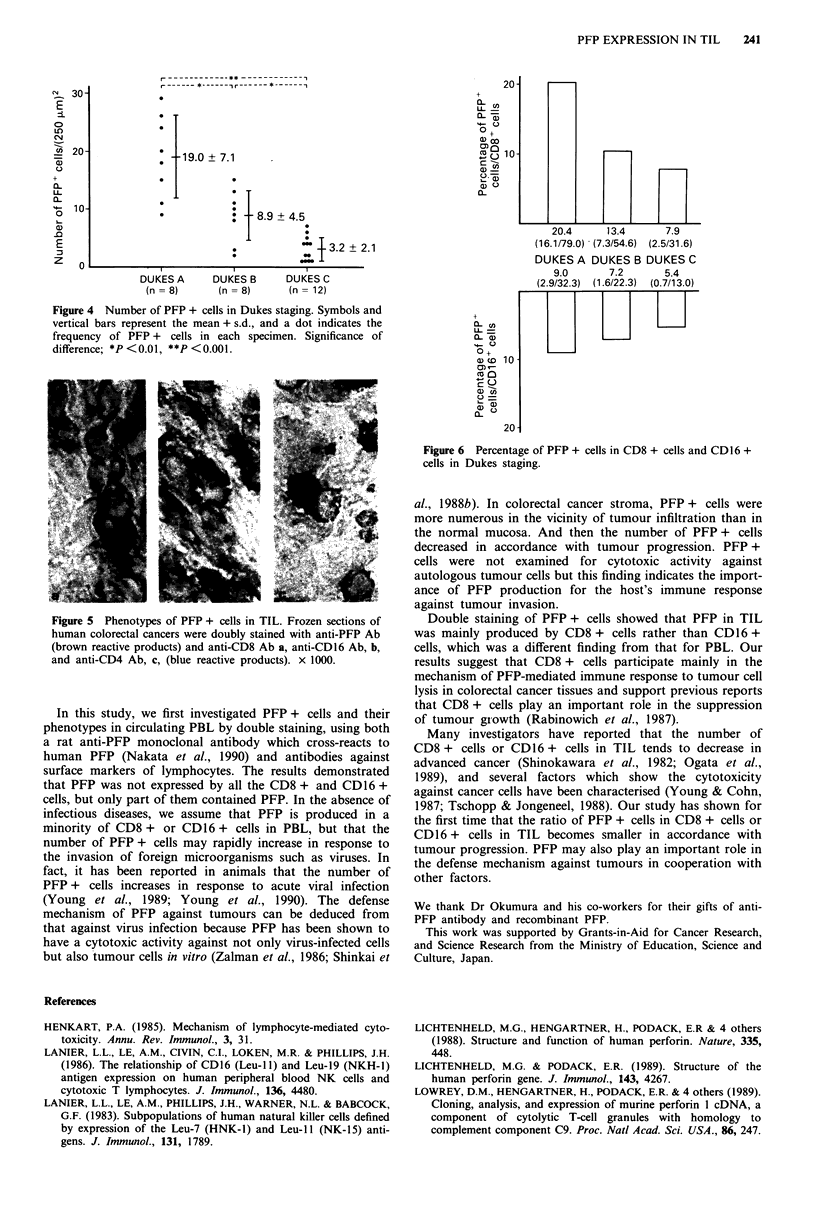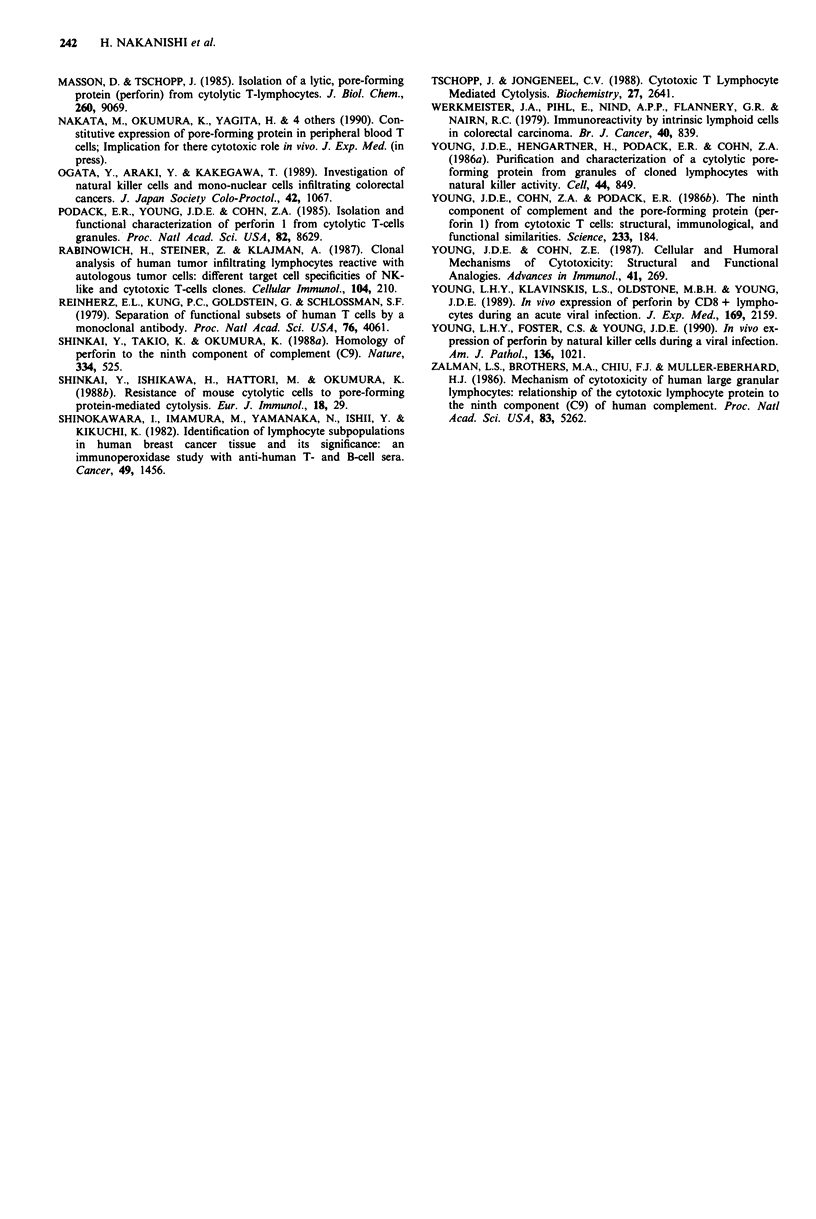# Perforin expression in lymphocytes infiltrated to human colorectal cancer.

**DOI:** 10.1038/bjc.1991.283

**Published:** 1991-08

**Authors:** H. Nakanishi, T. Monden, H. Morimoto, T. Kobayashi, T. Shimano, T. Mori

**Affiliations:** Department of Surgery II, Osaka University Medical School, Japan.

## Abstract

**Images:**


*Br. J. Cancer* (1991), **64**, 239–242 © Macmillan Press Ltd., 1991

### Perforin expression in lymphocytes infiltrated to human colorectal cancer

H. Nakanishi, T. Monden, H. Morimoto, T. Kobayashi, T. Shimano & T. Mori

*Department of Surgery II, Osaka University Medical School, 1-1-50 Fukushima, Fukushima-ku, Osaka 553, Japan.*

**Summary** Perforin (PFP) is a cytotoxic protein released from killer cells. PFP immunoreactivity in human peripheral blood lymphocytes (PBL) and tumour infiltrating lymphocytes (TIL) was investigated immunocytochemically with the aid of an anti-PFP monoclonal antibody. PFP was detected in the cytoplasm of 10% of PBL. We performed a double staining of PFP + cells with Leu11b/CD16, Leu2a/CD8, or Leu3a/CD4 and showed that PFP was produced by 9% of CD8 + cells and 18% of CD16 + cells but not by CD4 + cells. In 28 colorectal cancer tissues, PFP immunoreactivity was observed in the lymphocytes infiltrating to the tumour stroma. The PFP + cells were most numerous in Dukes A and decreased in number according to the progression of tumours. The PFP + cells in TIL exhibited the same phenotypes as those in PBL but the PFP + cells were more numerous in CD8 + cells than in CD16 + cells at all stages. This study represents the first evidence that PFP is mainly secreted from CD8 + cells in tumour tissues. It is hypothesised that the decrease in the number of PFP + cells in accordance with tumour progression may reflect the suppression of the host's local immunity.

Perforin (PFP) is a cytotoxic protein contained in the cytoplasmic granules of killer cells (Masson & Tschopp, 1985; Podack *et al.*, 1985; Young *et al.*, 1986*a*). PFP released from killer cells forms pores on the target cell membrane and induces cell lysis (Henkart, 1985; Zalman *et al.*, 1986; Young *et al.*, 1986*b*). Recent success in the cloning of PFP cDNA (Shinkai *et al.*, 1988*a*; Lichtenheld *et al.*, 1988; Lichtenheld & Podack, 1989; Lowrey *et al.*, 1989) has allowed the examination of mRNA expression of PFP in killer cells. In the T cell lines, PFP gene expression was ascertained in the cytotoxic T lymphocytes (CTL) and natural killer (NK) cells, but not in helper T (Th) cells (Shinkai *et al.*, 1988*a*; Lichtenheld *et al.*, 1988).

The role of tumour infiltrating lymphocytes (TIL) in the defense mechanism of a tumour-bearing host has been thoroughly reported (Werkmeister *et al.*, 1979). It is known that CTL and NK cells directly kill tumour cells (Reinherz *et al.*, 1979; Lanier *et al.*, 1983; Lanier *et al.*, 1986). In human cancer tissues, PFP may be derived from CTL and NK cells and may play an important role in anti-tumour activity.

In this paper, we aimed to identify the phenotypes of human peripheral blood lymphocytes (PBL) and TIL, which produce PFP. We employed a double staining method using both anti-PFP antibody and antibodies against surface markers of lymphocytes. Furthermore, we investigated the relation of PFP and tumour progression in human colorectal cancer stroma.

### Materials and methods

### *Cell preparation*

Human PBL were prepared from healthy persons. Briefly, PBL were recovered from the intermediate layer of Lymphocyte Separate Medium and suspended in RPMI 1640. The cells were cytocentrifuged (400 r.p.m.; 9 min), fixed in 4°C acetone for 10 min, in 4% paraformaldehyde for 10 min at room temperature, and then transferred to 0.05 mol $l^{-1}$ Tris -HCl (pH 7.6).

### *Tumour tissue preparation*

Fresh specimens of 28 human colorectal cancer tissues were frozen in OCT compound and kept frozen at − 80°C. Four micron-thick sections were prepared from each sample and fixed in acetone and 4% paraformaldehyde as described under. These samples were analysed with immunohistochemical staining for PFP and with haematoxylin and eosin staining for morphology. The clinicopathologic analyses were carried out according to the criteria of Dukes classification. Eight of the 28 subjects were early cancer cases, eight were advanced without metastasis, and twelve were advanced with metastasis. Based on these findings, eight of the cases were classified as Dukes A, eight as Dukes B and 12 as Dukes C. Histological examination revealed that all of the tumours were adenocarcinomas and showed a normal differentiation distribution (17 cases; well differentiated, ten cases; moderately differentiated one case; poorly differentiated). Normal colorectal tissues, 10 cm distant from the tumours, were used as controls.

### *Immunostaining*

*Single staining* A modification of the immunoglobulin enzyme bridge technique (ABC complex method) was used. Briefly, non specific binding was blocked by treatment with 2% (v/v) normal rabbit serum (Vector) for 30 min. Primary monoclonal antibody to recombinant PFP (rPFP), kindly provided by Dr Okumura (University of Juntendo Medical School), was applied to the sections at a dilution of 1:500, and incubated for 60 min at room temperature. After the sections were washed three times in Tris-HCl for 30 min, biotinylated second antibody of rabbit anti-rat IgG (mouse IgG adsorbed) (Vector) was applied at a dilution of 1:250, incubated for 30 min at room temperature, and washed three times in Tris-HCl. After quenching the endogenous peroxidase activity for 20 min in distilled water containing 0.1% NaN3 and 1% (v/v) hydrogen peroxidase, freshly prepared ABC complex (Vectastain R ABC Kit, Vector) was applied and followed by incubation for 30 min. After the excess complex was washed out, the localisation of PFP was visualised by incubating the sections for 5 min in freshly prepared Tris-HCl containing both 0.02% (w/v) 3.3′-diaminobenzidine tetrahydrochloride (Sigma, St Louis, MO) and 0.03% (v/v) hydrogen peroxide. The nuclei were counterstained with haematoxylin.

*Double staining* After blocking non specific binding by treatment with 2% (v/v) normal serum (Vector) for 30 min, primary monoclonal antibodies to Leu11b, Leu2a or Leu3a (Vector) were applied to each section at a dilution of 1:25, and incubated for 2 h at room temperature. After the sections were washed three times in Tris-HCl for 30 min, biotinylated second antibody of goat anti-mouse IgM for Leu11b (Vector) or horse anti-mouse IgG for Leu2a and Leu3a (rat IgG adsorbed) (Vector) was applied at a dilution

Correspondence: H. Nakanishi.
Received 19 November 1990; and in revised form 14 March 1991.

of 1:250, incubated for 30 min at room temperature and washed three times in Tris-HCl. After quenching the endogenous alkaline phosphatase activity in 1% (w/v) levamisol hydrochloride (Aldrich Chemical Company. Inc.) for 5 min, the sections were incubated in avidin-biotinylated alkaline phosphatase complex (Vector) for 30 min. After the excess complex was washed out, the localisation of CD16 +, CD8 + and CD4 + cells was visualised by incubating the sections for 10 min with an A.P. Substrate Kit 3, Blue (Maker Code SK 5000 300, Vector). The sections were then immunostained for PFP as described above. The sections were also counterstained with methylgreen.

### *Controls*

For negative controls, we prepared the antibody preabsorbed by rPFP: Wells of an immobilon ELISA titer plate (Dynatech-Logo) were incubated overnight with approximately 500 ng of rPFP in 50 microliters of PBS to bind rPFP to the plastic. Each well was then incubated with 100 microliters of anti-rPFP antibody at room temperature for 4 h. This adsorption procedure was repeated twice, and the absorbed antibody was used for immunostaining controls.

### *Calculation of PFP positive cells*

The numbers of PFP + cells infiltrated to cancer stromas were counted in three areas of 250 × 250 μ under high power magnification with an objective micrometer. The data were analysed for statistical significance with the non-parametric Welch test, and the results were considered significant if the *P* value was less than 0.05.

### Results

### *PFP + cells in PBL*

Specific PFP immunostaining was observed in the cytoplasm of lymphocytes (Figure 1). PFP was detected in large lymphocytes and accounted for about 10% of PBL. Preimmune rat serum or preabsorbed antibody used as a control did not yield specific staining. These control tests were repeated on the cytocentrifuged specimens of PBL with the same results. Double staining showed that 18% of CD16 + cells and 9% of CD8 + cells but none of the CD4 + cells produced PFP (Figure 2).

### *PFP +* cells in cancer stroma

Frozen sections of 28 colorectal cancers were evaluated for PFP specific immunostaining. Figure 3 shows the results obtained. PFP immunoreactivity was observed in the lymphocytes infiltrating to the tumour stroma, especially in the vicinity of tumour infiltration, but only a little in the normal epithelia. Preimmune rat serum used as a control did not yield specific staining. These control tests were repeated on sections of TIL with the same results.

The mean numbers of PFP + cells which were counted in three fields on each specimen were indicated with a dot in Figure 4. The number of PFP + cells in Dukes A was 19.0 ± 7.1 (mean ± s.d.), in Dukes B 8.9 ± 4.5 (mean ± s.d.), and in Dukes C 3.2 ± 2.1 (mean ± s.d.). The number of PFP + cells in Dukes A was significantly higher than in Dukes B ($P < 0.01$), and that in Dukes B significantly higher than in Dukes C ($P < 0.01$). Furthermore, the number in Dukes A was significantly much higher than in Dukes C ($P < 0.001$). The populations of PFP + cells were determined with double staining. A part of the CD16 + cells and CD8 + cells was doubly stained by anti PFP antibody but CD4 + cells were not doubly stained in TIL (Figure 5). PFP + cells in CD8 + and CD16 + cells were counted. The ratios of PFP + cells in CD8 + cells and in CD16 + cells were highest in Dukes A, while the number of PFP + cells decreased according to the progression of tumours. In all stages, the ratio of PFP + cells in CD8 + cells was higher than that in CD16 + cells (Figure 6).

**Figure 2** Phenotypes of PFP + cells in PBL. Human PBL were doubly stained with anti-PFP Ab (brown reactive products) and anti-CD8 Ab, **a**, anti-CD16 Ab, **b**, and anti-CD4 Ab, **c**, (blue reactive products). × 1000.

### Discussion

Northern blot analysis has confirmed that PFP is expressed by human CTL and NK cells *in vitro* (Shinkai *et al.*, 1988*a*; Lichtenheld *et al.*, 1988), but the expression of this protein *in vivo* has not yet been shown.

**Figure 1** PFP expression in PBL. Human PBL were stained with anti-PFP Ab. PFP immunoreactivity is observed in the cytoplasm of human PBL. × 800.

**Figure 3** PFP expression in TIL. Frozen sections of human colorectal cancers were stained with anti-PFP Ab. PFP immunoreactivity is observed in the lymphocytes infiltrated to human colorectal cancer. × 200.

**Figure 4** Number of PFP + cells in Dukes staging. Symbols and vertical bars represent the mean + s.d., and a dot indicates the frequency of PFP + cells in each specimen. Significance of difference; $^{*}P<0.01$, $^{**}P<0.001$.

**Figure 5** Phenotypes of PFP + cells in TIL. Frozen sections of human colorectal cancers were doubly stained with anti-PFP Ab (brown reactive products) and anti-CD8 Ab **a**, anti-CD16 Ab, **b**, and anti-CD4 Ab, **c**, (blue reactive products). × 1000.

**Figure 6** Percentage of PFP + cells in CD8 + cells and CD16 + cells in Dukes staging.

In this study, we first investigated PFP + cells and their phenotypes in circulating PBL by double staining, using both a rat anti-PFP monoclonal antibody which cross-reacts to human PFP (Nakata *et al.*, 1990) and antibodies against surface markers of lymphocytes. The results demonstrated that PFP was not expressed by all the CD8 + and CD16 + cells, but only part of them contained PFP. In the absence of infectious diseases, we assume that PFP is produced in a minority of CD8 + or CD16 + cells in PBL, but that the number of PFP + cells may rapidly increase in response to the invasion of foreign microorganisms such as viruses. In fact, it has been reported in animals that the number of PFP + cells increases in response to acute viral infection (Young *et al.*, 1989; Young *et al.*, 1990). The defense mechanism of PFP against tumours can be deduced from that against virus infection because PFP has been shown to have a cytotoxic activity against not only virus-infected cells but also tumour cells *in vitro* (Zalman *et al.*, 1986; Shinkai *et al.*, 1988*b*). In colorectal cancer stroma, PFP + cells were more numerous in the vicinity of tumour infiltration than in the normal mucosa. And then the number of PFP + cells decreased in accordance with tumour progression. PFP + cells were not examined for cytotoxic activity against autologous tumour cells but this finding indicates the importance of PFP production for the host's immune response against tumour invasion.

Double staining of PFP + cells showed that PFP in TIL was mainly produced by CD8 + cells rather than CD16 + cells, which was a different finding from that for PBL. Our results suggest that CD8 + cells participate mainly in the mechanism of PFP-mediated immune response to tumour cell lysis in colorectal cancer tissues and support previous reports that CD8 + cells play an important role in the suppression of tumour growth (Rabinowich *et al.*, 1987).

Many investigators have reported that the number of CD8 + cells or CD16 + cells in TIL tends to decrease in advanced cancer (Shinokawara *et al.*, 1982; Ogata *et al.*, 1989), and several factors which show the cytotoxicity against cancer cells have been characterised (Young & Cohn, 1987; Tschopp & Jongeneel, 1988). Our study has shown for the first time that the ratio of PFP + cells in CD8 + cells or CD16 + cells in TIL becomes smaller in accordance with tumour progression. PFP may also play an important role in the defense mechanism against tumours in cooperation with other factors.

We thank Dr Okumura and his co-workers for their gifts of anti-PFP antibody and recombinant PFP.

This work was supported by Grants-in-Aid for Cancer Research, and Science Research from the Ministry of Education, Science and Culture, Japan.

### References

HENKART, P.A. (1985). Mechanism of lymphocyte-mediated cytotoxicity. *Annu. Rev. Immunol.*, **3**, 31.

LANIER, L.L., LE, A.M., CIVIN, C.I., LOKEN, M.R. & PHILLIPS, J.H. (1986). The relationship of CD16 (Leu-11) and Leu-19 (NKH-1) antigen expression on human peripheral blood NK cells and cytotoxic T lymphocytes. *J. Immunol.*, **136**, 4480.

LANIER, L.L., LE, A.M., PHILLIPS, J.H., WARNER, N.L. & BABCOCK, G.F. (1983). Subpopulations of human natural killer cells defined by expression of the Leu-7 (HNK-1) and Leu-11 (NK-15) antigens. *J. Immunol.*, **131**, 1789.

LICHTENHELD, M.G., HENGARTNER, H., PODACK, E.R & 4 others (1988). Structure and function of human perforin. *Nature*, **335**, 448.

LICHTENHELD, M.G. & PODACK, E.R. (1989). Structure of the human perforin gene. *J. Immunol.*, **143**, 4267.

LOWREY, D.M., HENGARTNER, H., PODACK, E.R. & 4 others (1989). Cloning, analysis, and expression of murine perforin 1 cDNA, a component of cytolytic T-cell granules with homology to complement component C9. *Proc. Natl Acad. Sci. USA.*, **86**, 247.

MASSON, D. & TSCHOPP, J. (1985). Isolation of a lytic, pore-forming protein (perforin) from cytolytic T-lymphocytes. *J. Biol. Chem.*, **260**, 9069.

NAKATA, M., OKUMURA, K., YAGITA, H. & 4 others (1990). Constitutive expression of pore-forming protein in peripheral blood T cells; Implication for there cytotoxic role *in vivo*. *J. Exp. Med.* (in press).

OGATA, Y., ARAKI, Y. & KAKEGAWA, T. (1989). Investigation of natural killer cells and mono-nuclear cells infiltrating colorectal cancers. *J. Japan Society Colo-Proctol.*, **42**, 1067.

PODACK, E.R., YOUNG, J.D.E. & COHN, Z.A. (1985). Isolation and functional characterization of perforin 1 from cytolytic T-cells granules. *Proc. Natl Acad. Sci. USA*, **82**, 8629.

RABINOWICH, H., STEINER, Z. & KLAJMAN, A. (1987). Clonal analysis of human tumor infiltrating lymphocytes reactive with autologous tumor cells: different target cell specificities of NK-like and cytotoxic T-cells clones. *Cellular Immunol.*, **104**, 210.

REINHERZ, E.L., KUNG, P.C., GOLDSTEIN, G. & SCHLOSSMAN, S.F. (1979). Separation of functional subsets of human T cells by a monoclonal antibody. *Proc. Natl Acad. Sci. USA*, **76**, 4061.

SHINKAI, Y., TAKIO, K. & OKUMURA, K. (1988*a*). Homology of perforin to the ninth component of complement (C9). *Nature*, **334**, 525.

SHINKAI, Y., ISHIKAWA, H., HATTORI, M. & OKUMURA, K. (1988*b*). Resistance of mouse cytolytic cells to pore-forming protein-mediated cytolysis. *Eur. J. Immunol.*, **18**, 29.

SHINOKAWARA, I., IMAMURA, M., YAMANAKA, N., ISHII, Y. & KIKUCHI, K. (1982). Identification of lymphocyte subpopulations in human breast cancer tissue and its significance: an immunoperoxidase study with anti-human T- and B-cell sera. *Cancer*, **49**, 1456.

TSCHOPP, J. & JONGENEEL, C.V. (1988). Cytotoxic T Lymphocyte Mediated Cytolysis. *Biochemistry*, **27**, 2641.

WERKMEISTER, J.A., PIHL, E., NIND, A.P.P., FLANNERY, G.R. & NAIRN, R.C. (1979). Immunoreactivity by intrinsic lymphoid cells in colorectal carcinoma. *Br. J. Cancer*, **40**, 839.

YOUNG, J.D.E., HENGARTNER, H., PODACK, E.R. & COHN, Z.A. (1986*a*). Purification and characterization of a cytolytic pore-forming protein from granules of cloned lymphocytes with natural killer activity. *Cell*, **44**, 849.

YOUNG, J.D.E., COHN, Z.A. & PODACK, E.R. (1986*b*). The ninth component of complement and the pore-forming protein (perforin 1) from cytotoxic T cells: structural, immunological, and functional similarities. *Science*, **233**, 184.

YOUNG, J.D.E. & COHN, Z.E. (1987). Cellular and Humoral Mechanisms of Cytotoxicity: Structural and Functional Analogies. *Advances in Immunol.*, **41**, 269.

YOUNG, L.H.Y., KLAVINSKIS, L.S., OLDSTONE, M.B.H. & YOUNG, J.D.E. (1989). *In vivo* expression of perforin by CD8 + lymphocytes during an acute viral infection. *J. Exp. Med.*, **169**, 2159.

YOUNG, L.H.Y., FOSTER, C.S. & YOUNG, J.D.E. (1990). *In vivo* expression of perforin by natural killer cells during a viral infection. *Am. J. Pathol.*, **136**, 1021.

ZALMAN, L.S., BROTHERS, M.A., CHIU, F.J. & MULLER-EBERHARD, H.J. (1986). Mechanism of cytotoxicity of human large granular lymphocytes: relationship of the cytotoxic lymphocyte protein to the ninth component (C9) of human complement. *Proc. Natl Acad. Sci. USA*, **83**, 5262.